# Fullerene-Functionalized Cellulosic Hydrogel Biosensor with Bacterial Turn-on Fluorescence Response Derived from Carboxymethyl Cellulose for Intelligent Food Packaging with DFT Calculations and Molecular Docking

**DOI:** 10.3390/gels11050329

**Published:** 2025-04-28

**Authors:** Hebat-Allah S. Tohamy

**Affiliations:** Cellulose and Paper Department, National Research Centre, 33 El Bohouth Str., Dokki Giza P.O. 12622, Egypt; hebasarhan89@yahoo.com or hs.tohamy@nrc.sci.eg

**Keywords:** turn-on fluorescence response, biosensor, cellulosic hydrogel, bacterial detection, antibacterial activity, nitrogen-doped fullerenes

## Abstract

This study reports the synthesis and characterization of a novel carboxymethyl cellulose–N-fullerene–g-poly(co-acrylamido-2-methyl-1-propane sulfonic acid) (CMC–N-fullerene–AMPS) hydrogel for potential application in biosensing within food packaging. The hydrogel was synthesized via free radical polymerization and characterized using FTIR, SEM, and fluorescence microscopy. FTIR analysis confirmed the successful grafting of AMPS and incorporation of N-fullerenes, indicated by characteristic peaks and a shift in the N–H/O–H stretching frequency. Density Functional Theory (DFT) calculations revealed that the CMC–N-fullerene–AMPS hydrogel exhibited higher stability and a lower band gap energy (0.0871 eV) compared to the CMC–AMPS hydrogel, which means a high reactivity of CMC–N-fullerene–AMPS. The incorporation of N-fullerenes significantly enhanced the hydrogel’s antibacterial activity, demonstrating a 22 mm inhibition zone against *E. coli* and a 24 mm zone against *S. aureus*, suggesting potential for active food packaging applications. Critically, the hydrogel displayed a unique “turn-on” fluorescence response in the presence of bacteria, with distinct color changes observed upon interaction with *E. coli* (orange-red) and *S. aureus* (bright green). This fluorescence enhancement, coupled with the porous morphology observed via SEM (pore size 377–931 µm), suggests the potential of this hydrogel as a sensing platform for bacterial contamination within food packaging. These combined properties of enhanced antibacterial activity and a distinct, bacteria-induced fluorescence signal make the CMC–N-fullerene–AMPS hydrogel a promising candidate for developing intelligent food packaging materials capable of detecting bacterial spoilage.

## 1. Introduction

The sheer scale of food waste globally presents a critical challenge with wide-ranging repercussions. A substantial portion of food intended for human consumption, roughly a third, or about 1.3 billion tons each year, is either lost or wasted. This loss and waste occur at every stage, from farm production and processing to retail sales and household consumption. In developing nations, much of the loss occurs during production, often due to inefficient harvesting methods, inadequate storage, and limited market access. Conversely, in wealthier nations, waste is more common at the retail and consumer levels, driven by factors like cosmetic imperfections in produce, purchasing more than needed, and improper food storage leading to spoilage [1,2,3]. The consequences of this waste are considerable. Food waste is responsible for a significant portion of global greenhouse gas emissions, estimated at 8–10%, making it a key contributor to climate change [4,5,6]. Furthermore, discarded food frequently ends up in landfills, where it decomposes and generates methane, a powerful greenhouse gas [7,8]. The economic implications are also significant. Food waste represents a substantial loss of resources and income for farmers, businesses, and individuals, affecting both rich and poor countries [9,10]. While food packaging plays a vital role in protecting and preserving food, its current forms often present challenges that worsen the issue of food waste. A key concern is the lack of biodegradability in many commonly used materials, which significantly contributes to environmental pollution. For instance, widely adopted plastics like polyethylene (PE) and polypropylene (PP), favored for their affordability and adaptability, can persist for centuries in the environment [11,12]. This slow decomposition results in the build-up of waste in landfills and oceans, negatively impacting ecosystems and potentially introducing microplastics into the food chain. Moreover, the recycling of these plastics, even when feasible, can require substantial energy input and may yield materials with diminished quality, restricting their ability to be reused effectively [13,14]. Smart packaging marks a major leap forward in food technology, providing innovative solutions to the intertwined challenges of food safety and waste. Moving beyond the passive role of traditional packaging, smart packaging actively engages with food and its surroundings, offering insights into quality, freshness, and safety. This is accomplished by incorporating technologies like sensors, indicators, and antimicrobial agents. These advancements offer significant promise for minimizing food waste by prolonging shelf life, delivering real-time data on food condition, and inhibiting spoilage [15,16]. The unique characteristics of fullerenes, particularly C60, including their spherical shape, extensive surface area, and ability to accept electrons, position them as promising candidates for revolutionizing food packaging [17,18,19,20]. These properties endow them with potent antimicrobial capabilities [21,22]. When integrated into food packaging materials, fullerenes can effectively extend the shelf life of perishable foods by inhibiting microbial growth and safeguarding against oxidative spoilage [23,24,25,26]. This translates to a reduction in food waste and a significant enhancement in food safety [25,27]. Conventional fullerene synthesis methods, such as the energy-intensive and environmentally detrimental arc-discharge technique, present significant drawbacks [27,28,29,30]. Conversely, utilizing sugarcane bagasse (SB), a readily available and renewable byproduct of the sugar industry, offers a sustainable alternative for fullerene production [31,32,33]. Transforming this agricultural waste into valuable materials like fullerenes not only generates economic advantages but also mitigates environmental pollution [34,35,36,37]. The production of fullerenes from SB typically entails a multi-step process encompassing carbonization, purification, and subsequent processing to isolate C60. Its environmental and economic benefits make it a highly promising avenue for sustainable fullerene production [38]. Fullerenes exhibit inherent fluorescence properties that make them valuable for developing innovative food packaging sensors [39]. One key characteristic is their ability to undergo thermally activated delayed fluorescence (TADF). In TADF, upon absorbing light, the fullerene molecule enters an excited state [40,41,42,43]. Instead of immediately emitting fluorescence, it undergoes a thermally activated process, transitioning to a lower energy state before emitting light. This delayed fluorescence emission provides a unique mechanism for sensing applications [44,45]. Furthermore, fullerenes can act as efficient fluorescence quenchers [40,46]. When a fluorescent molecule is in close proximity to a fullerene, the energy of the excited state of the fluorescent molecule can be transferred to the fullerene, leading to a decrease in the fluorescence intensity of the molecule [46,47]. This fluorescence-quenching phenomenon can be effectively utilized to create sensors that detect the presence of specific analytes in food, such as pathogens or toxins [48,49,50]. By strategically incorporating fullerenes into food packaging materials and designing appropriate sensing mechanisms, it becomes possible to develop systems that can detect the presence of contaminants or spoilage in food products. These sensors have the potential to provide real-time information about the safety and quality of food, ultimately contributing to a reduction in food waste and an improvement in consumer health.

Hydrogels, with their high water content, responsiveness, and safety, are in high demand for biological applications [51,52,53]. pH-sensitive hydrogels offer enhanced controllability [15]. Incorporating 2-Acrylamido-2-methyl-1-propane-sulfonic acid (AMPS) into carboxymethyl cellulose (CMC) enables the creation of hydrogels with enhanced properties for food packaging [54,55]. The sulfonic acid groups of AMPS impart hydrophilicity and an ionic character, while the acrylamide moiety facilitates crosslinking and the formation of a robust three-dimensional network [56,57,58,59]. These combined features position AMPS-modified CMC hydrogels as promising candidates for developing innovative and sustainable food packaging solutions. The microwave method for preparing CMC offers significant advantages over conventional methods. It is significantly faster, reducing reaction times considerably compared to traditional heating techniques. This expedited process translates to increased efficiency and reduced energy consumption, making it a more environmentally friendly approach. Moreover, microwave irradiation often results in improved product quality and reduced byproduct formation, further enhancing the sustainability and overall appeal of this method for CMC production [16,60]. This article presents a green and sustainable method for synthesizing antimicrobial/sensing carboxymethyl cellulose–fullerene–g-poly(co-acrylamido-2-methyl-1-propane sulfonic acid) hydrogel. The prepared carboxymethyl cellulose–fullerene–g-poly(co-acrylamido-2-methyl-1-propane sulfonic acid) was characterized using FTIR, SEM, DFT, molecular docking, and antimicrobial activity assays.

## 2. Results and Discussion

### 2.1. Proposed Mechanism of Carboxymethyl Cellulose–N-fullerene–g-poly(co-acrylamido-2-methyl-1-propane Sulfonic Acid) Hydrogel Formation and the Swelling Study

**Figure 1:**
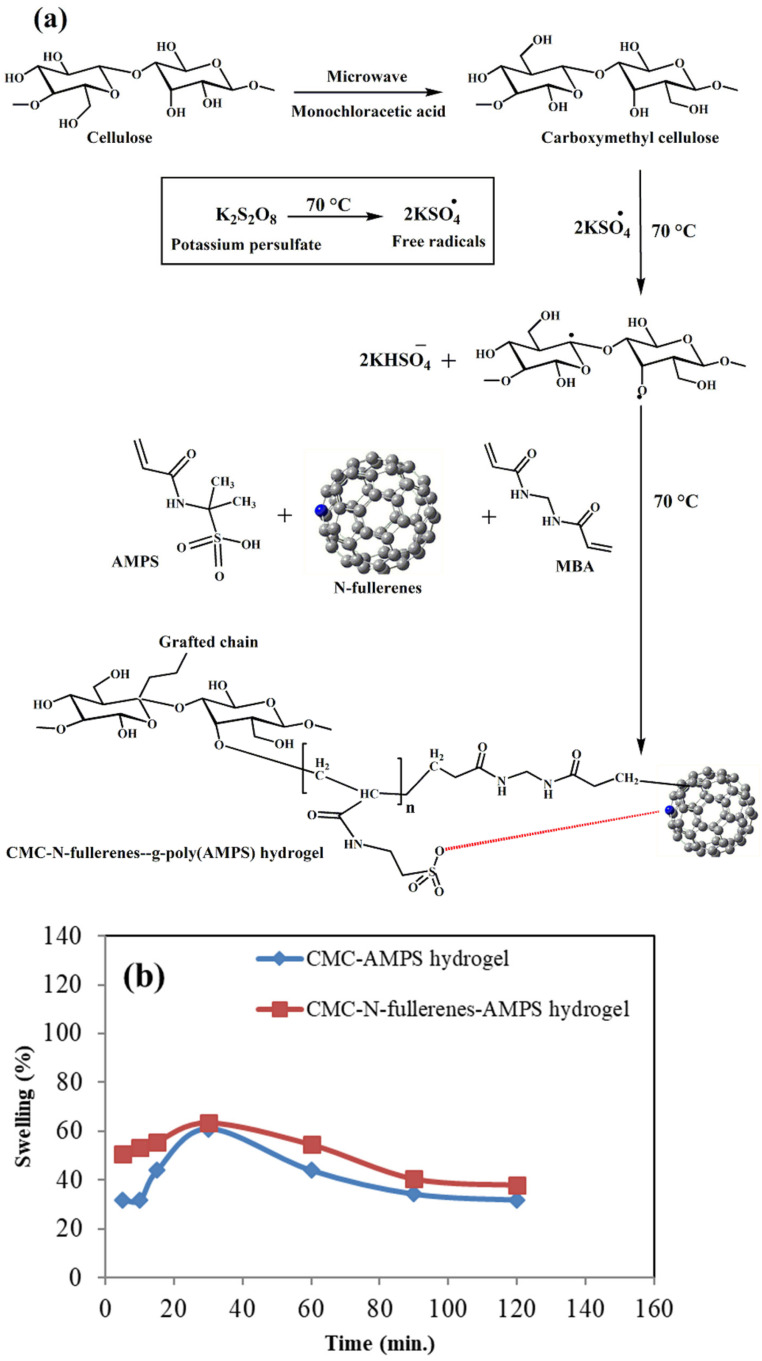
(**a**) Reaction mechanism for synthesizing carboxymethyl cellulose–N-fullerene–g-poly(co-acrylamido-2-methyl-1-propane sulfonic acid) hydrogel and (**b**) the swelling study of CMC–AMPS hydrogel and CMC–N-fullerene–AMPS hydrogel.

When heated, the KPS initiator breaks down into sulfate anion radicals (SO_4_^•^) [61,62,63]. The grafting and crosslinking mechanism is depicted in Figure 1. Initiation begins with the thermal decomposition of KPS, producing sulfate anion radicals (SO_4_^•^). These radicals abstract hydrogen atoms from CMC’s hydroxyl groups, generating alkoxy radicals along the CMC backbone. These alkoxy radicals then react with adjacent AMPS monomers, initiating polymerization. The growing polymer chains subsequently transfer free radicals to N-fullerene molecules, promoting further chain propagation and grafting. Simultaneously, the crosslinking agent, MBA, reacts with the propagating polymer chains via its vinyl groups, leading to the formation of a crosslinked copolymer network [56,64].

The swelling behavior of both hydrogels exhibited an initial increase within the first 30 min, with the CMC–AMPS hydrogel swelling from 31.70% to 60.97% and the CMC–N-fullerene–AMPS hydrogel swelling from 50.63% to 63.29%. This initial increase is likely due to the hydrophilic nature of the AMPS groups and water molecules penetrating the hydrogel matrix. However, after 60 min, a decrease in swelling was observed for both hydrogels. The subsequent decrease in swelling after 60 min could indicate possible changes in the hydrogel structure; e.g., further crosslinking occurring within the hydrogel network, or perhaps some breakdown of the network. This change in crosslinking density over time may suggest that the hydrogel network is starting to degrade.

### 2.2. Fourier Transform Infrared Spectroscopy (FTIR) Spectra

The prepared cellulose and CMC displayed peaks between 3221–3344 cm^−1^, 1450–1455 cm^−1^, 1315–1386 cm^−1^, 1029–1037 cm^−1^, and 882–896 cm^−1^, which are related to O–H, C–O bending, C–H bending, the C–O–C of pyranose ring vibration, and β-glycosidic linkage, respectively [65,66,67,68,69]. The additional peak at 1745 cm^−1^ in CMC is related to the C=O peak, while the N–fullerenes showed peaks at 3461 cm^−1^ (N–H), 3207 cm^−1^ (O–H), 1662 cm^−1^ (C=O), 1386 cm^−1^ (O=C–O), 1087 cm^−1^ (C–O–C), and 657 cm^−1^ (C–N) [70,71].

**Figure 2:**
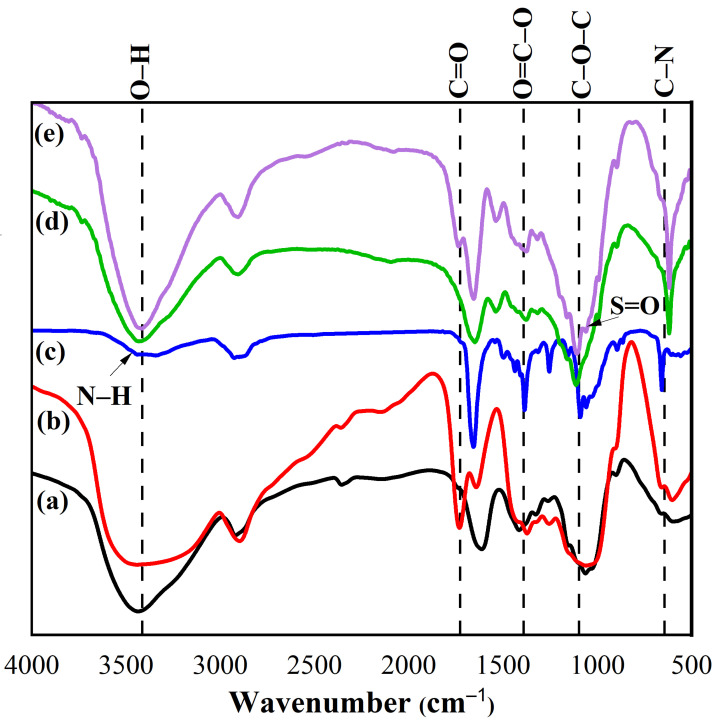
FTIR spectra of (**a**) cellulose, (**b**) CMC, (**c**) N-fullerenes, (**d**) CMC–AMPS hydrogel, and (**e**) CMC–N-fullerene–AMPS hydrogel.

The prepared CMC–AMPS and CMC–N-fullerene–AMPS hydrogels showed the same peaks of CMC with additional peaks between 986 and 1076 cm^−1^ (S=O), which proves the AMPS grafting (Figure 2) [56]. From FTIR observations, we found that the broad peak of N–H/O–H groups was shifted from a high value (3431 cm^−1^) for the CMC–AMPS hydrogel to a lower value (i.e., 3406 cm^−1^) for the CMC–N-fullerene–AMPS hydrogel, indicating strong H bonding between the CMC–N-fullerene–AMPS hydrogel compared to the CMC–AMPS hydrogel due to the incorporation of N–fullerenes [38,72].

### 2.3. DFT Calculations

**Figure 3:**
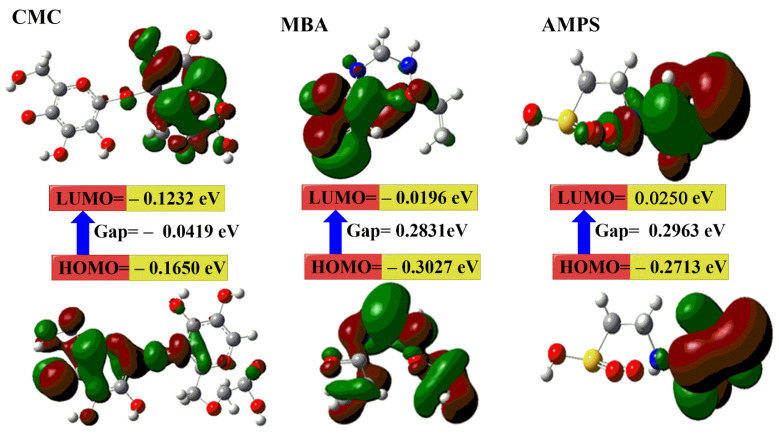

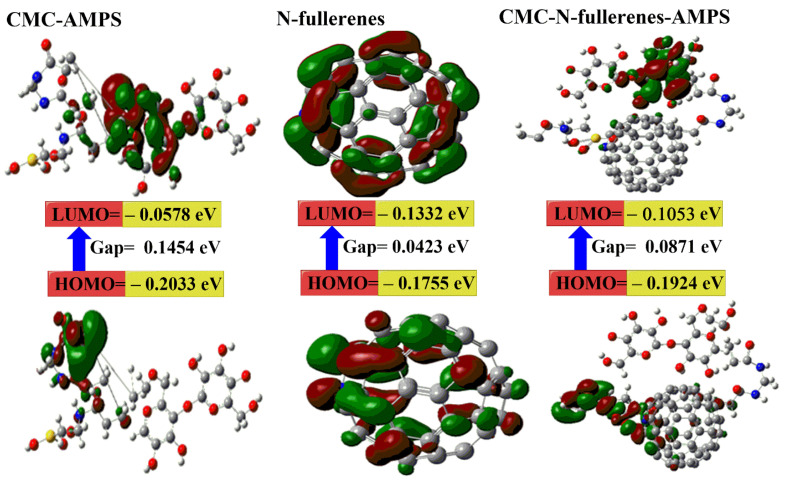
The gap energies (HOMO–LUMO) (eV) were calculated for the hydrogels using DFT B3LYP/6–31G (d), as was the molecular orbital interaction between CMC, MBA, AMPS, CMC–AMPS hydrogel, N-fullerenes, and CMC–N-fullerene–AMPS hydrogel.

To gain deeper insights into the structural and electronic properties of the synthesized hydrogels and to understand the impact of N-fullerene incorporation on their stability and reactivity, Density Functional Theory (DFT) calculations were performed. From Figure 3 and Table 1, the results show the following:

a. The μ and ω for the CMC–N-fullerene–AMPS hydrogel (i.e., 41.321 Debye and 0.2543 eV) is higher than the CMC–AMPS hydrogel (i.e., 24.309 Debye and 0.1172 eV) because of the presence of N-fullerenes [16,73,74].
b. The calculated E_g_ for the CMC–N-fullerene–AMPS hydrogel is the lowest (i.e., 0.0871 eV) compared to the CMC–AMPS hydrogel (i.e., 0.1454 eV), which in turn proves the strong chemical reaction between CMC, MBA, AMPS, and N-fullerenes in the CMC–N-fullerene–AMPS hydrogel compared to the CMC–AMPS hydrogel due to the presence of N-fullerenes [75,76].
c. The E_T_ of the CMC–N-fullerene–AMPS hydrogel (−5158.364 au) is lower than the CMC–AMPS hydrogel (−2741.402 au), which means that the formation of the CMC–N-fullerene–AMPS hydrogel releases energy. This released energy makes it harder to break the bonds in the CMC–N-fullerene–AMPS hydrogel [15,72].
d. The CMC–N-fullerene–AMPS hydrogel is much softer than the CMC–AMPS hydrogel, which is a good approximation of the strong energy changes between the donor (HOMO) and acceptor (LUMO) in the CMC–N-fullerene–AMPS hydrogel [75].

These DFT findings provide theoretical support for the enhanced properties observed experimentally. For instance, the lower band gap energy may contribute to the enhanced fluorescence or antibacterial activity (this will be discussed later). This suggests that N-fullerenes play a crucial role in modulating the electronic and structural characteristics of the hydrogel, leading to the observed improvements in performance.

### 2.4. Antibacterial Activity and Molecular Docking Study

**Figure 4:**
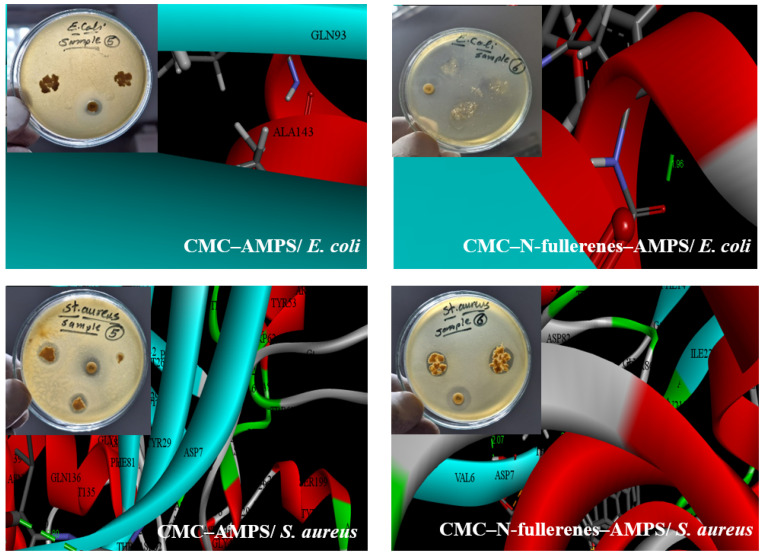
The antimicrobial and biological activity of CMC–AMPS hydrogel (denoted as 5) and CMC–N-fullerene–AMPS hydrogel (denoted as 6) as ligands against *Escherichia coli* PDB (3ZH7), and *Staphylococcus aureus* PDB (4QLO).

Our investigation demonstrated that the incorporation of N-fullerenes into the CMC–AMPS hydrogel significantly enhanced its antibacterial properties against both Gram-negative (*Escherichia coli*) and Gram-positive (*Staphylococcus aureus*) bacteria. While CMC–AMPS hydrogel exhibited no inhibition against *E. coli*, the CMC–N-fullerene–AMPS hydrogel displayed a substantial inhibition zone of 22 mm. Similarly, for *S. aureus*, the CMC–N-fullerene–AMPS hydrogel demonstrated a larger inhibition zone (24 mm) compared to CMC–AMPS hydrogel (15 mm). This enhanced antibacterial activity against Gram-positive bacteria is likely attributed to the presence of oxygen (O), nitrogen (N), and sulfur (S) functionalities within the CMC–N-fullerene–AMPS hydrogel (Figure 4) [77,78,79,80,81]. These functional groups are capable of interacting with key bacterial cell components, such as lipids, proteins, and DNA/RNA, through various mechanisms, including hydrogen bonding, π–π interactions, and electrostatic interactions [82,83]. Furthermore, the presence of N-fullerenes within the hydrogel may contribute to the observed antibacterial effect by regulating the generation of reactive oxygen species, potentially derived from bacterial surface proteins [84,85,86]. These findings were aligned with the docking results where the N–CDs showed binding with proteins with bond lengths of 0, 1.96, 2.80, and 2.07 A° for CMC–AMPS hydrogel/*Escherichia coli*, CMC–N-fullerene–AMPS hydrogel/*Escherichia coli*, CMC–AMPS hydrogel/*Staphylococcus aureus*, and CMC–N-fullerene–AMPS hydrogel/*Staphylococcus aureus*, respectively. Shorter bond lengths between the ligand and the protein result in a more reactive ligand. This increased reactivity enables the ligand to form a stronger bond (chelate) with the protein, potentially impairing its function and killing the microbe [15,16].

### 2.5. Mechanism of Bacterial-Induced Fluorescence Enhancement After Bacterial Contact

**Figure 5:**
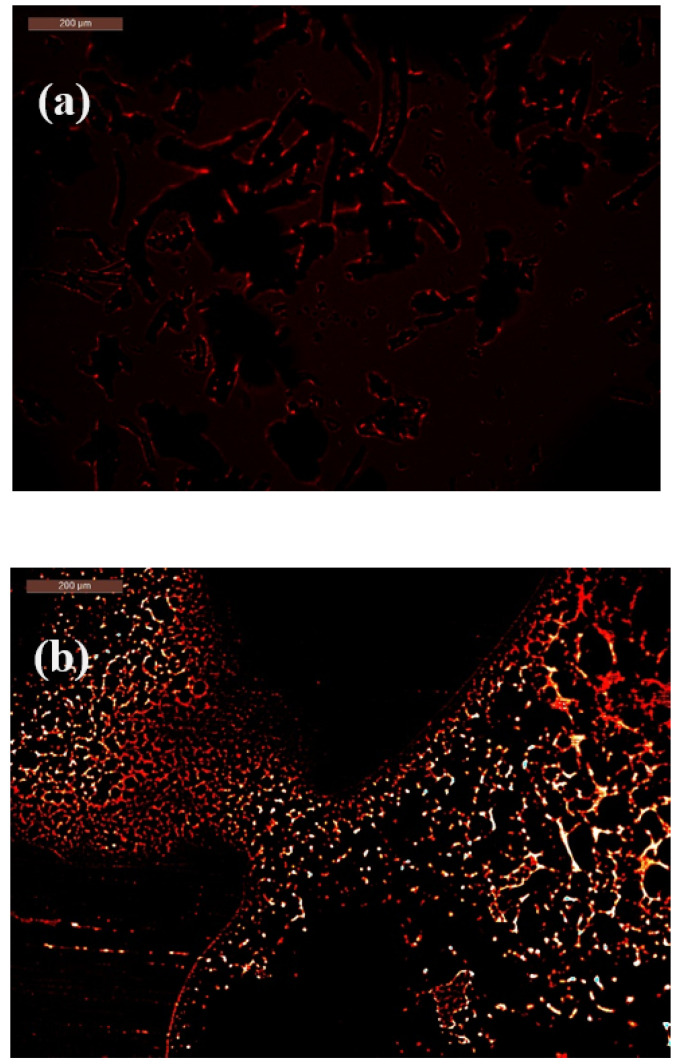

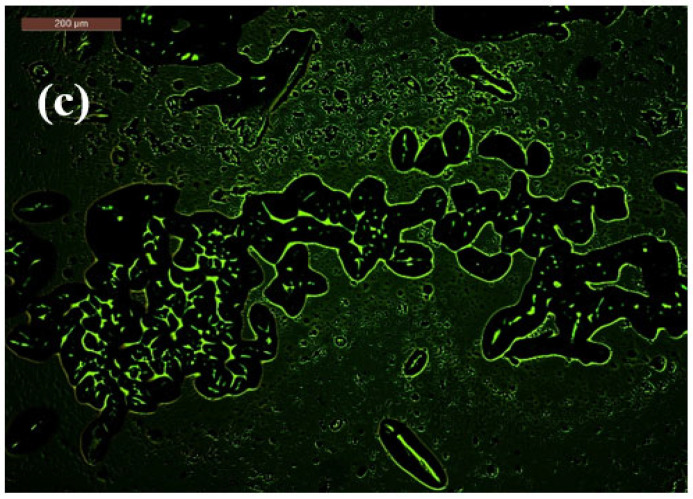
Fluorescence microscope for (**a**) CMC–N-fullerene–AMPS hydrogel before bacteria, (**b**) CMC–N-fullerene–AMPS hydrogel/*Escherichia coli*, and (**c**) CMC–N-fullerene–AMPS hydrogel/*Staphylococcus aureus*.

Initially, the CMC–N-fullerene–AMPS hydrogel appeared dark red. However, upon exposure to *E. coli*, a noticeable shift towards a brighter orange-red color was observed, accompanied by a broader color distribution. In contrast, interaction with *S. aureus* led to a more pronounced and uniform bright green coloration, demonstrating the “turn-on” fluorescence response of the hydrogel in the presence of both Gram-negative and Gram-positive bacteria (Figure 5).

The “turn-on” fluorescence observed in the CMC–N-fullerene–AMPS hydrogel upon bacterial contact suggests an intriguing interaction. Several mechanisms could be responsible for this phenomenon. One possibility is Fluorescence Resonance Energy Transfer (FRET). FRET is a distance-dependent energy transfer process where an excited donor fluorophore transfers energy to an acceptor chromophore. In this scenario, bacterial components, such as proteins or DNA, could act as energy acceptors. Upon bacterial interaction, energy transfer might occur from N-fullerenes within the hydrogel to these bacterial components, leading to an increase in fluorescence emission [87,88,89,90,91]. Another possible mechanism involves environmental changes induced by a bacterial metabolism [92,93]. Bacterial activity could alter the local environment of the hydrogel, such as pH or ionic strength. These changes could subsequently influence the electronic structure of the N-fullerenes, leading to enhanced fluorescence. Furthermore, direct N-fullerene–bacteria interactions cannot be ruled out. The N-fullerenes within the hydrogel might interact directly with bacterial components, potentially causing conformational changes in the N-fullerenes and consequently increasing their fluorescence quantum yield.

While Tohamy et al. observed a red-to-blue fluorescence shift with *E. coli* and a red emission with *S. aureus* using CMC@Ca-N-CDs, our CMC–N-fullerene–AMPS hydrogel exhibited a distinct “turn-on” fluorescence response, shifting from dark red to brighter orange-red with *E. coli* and to bright green with *S. aureus* [16]. A key advantage of our CMC–N-fullerene–AMPS hydrogel is the significant enhancement of fluorescence upon bacterial interaction. Unlike the subtle color changes reported by Tohamy et al., our system exhibited a dramatic increase in fluorescence intensity, transitioning from a dark red to bright orange-red with *E. coli* and bright green with *S. aureus*, suggesting a more sensitive detection mechanism.

### 2.6. Morphological Observations

**Figure 6:**
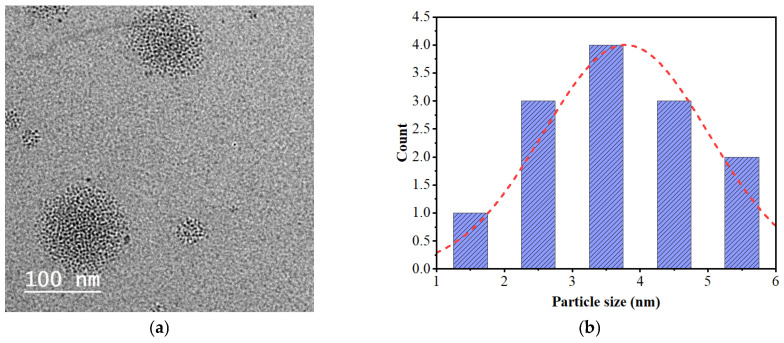

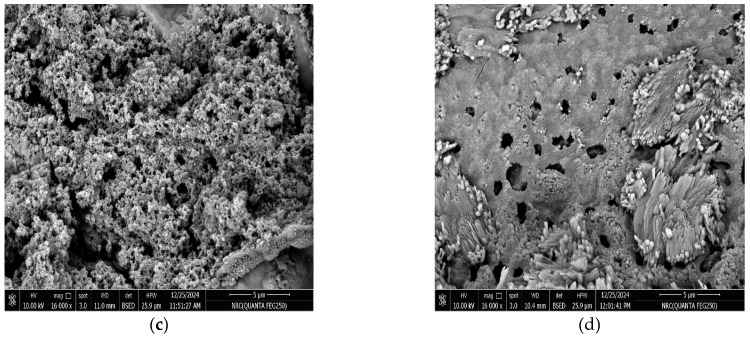
(**a**) TEM analysis for N-fullerenes, (**b**) particle size distribution for N-fullerenes, (**c**) SEM analysis for CMC–AMPS hydrogel, and (**d**) SEM analysis for CMC–N-fullerene–AMPS hydrogel.

TEM analysis of N-fullerenes illustrates big bucky ball-shaped carbon dots composed of individual carbon nanodots with a diameter ranging between ~1.85 and 4.93 nm (Figure 6a).

Figure 6b,c illustrates the surface morphology of the CMC–AMPS hydrogel and the CMC–N-fullerene–AMPS hydrogel. The CMC–AMPS hydrogel showed a porous-shaped film with a pore size ranging between 309–930 µm, while the CMC–N-fullerene–AMPS hydrogel showed a porous shape with flowery structures due to the presence of N-fullerenes. The porosity for the CMC–N-fullerene–AMPS hydrogel is equal to 377–931 µm. The porous structure of the CMC–N-fullerene–AMPS hydrogel enhances the interaction between the MCD sensor and its surroundings. This increased accessibility results in improved sensitivity and faster response times.

## 3. Conclusions

In conclusion, this study successfully synthesized a novel carboxymethyl cellulose–N-fullerene–g-poly(co-acrylamido-2-methyl-1-propane sulfonic acid) (CMC–N-fullerene–AMPS) hydrogel via a free radical polymerization method. FTIR analysis confirmed the successful grafting of AMPS and incorporation of N-fullerenes onto the CMC backbone, evidenced by characteristic S=O peaks and a shift in the N–H/O–H peak from 3431 cm^−1^ in CMC–AMPS to 3406 cm^−1^ in CMC–N-fullerene–AMPS, indicating strong hydrogen bonding. DFT calculations further corroborated the successful synthesis, demonstrating that the CMC–N-fullerene–AMPS hydrogel exhibited higher stability (μ = 41.321 Debye), a lower band gap energy (E_g_ = 0.0871 eV), and greater energy release upon formation (E_T_ = −5158.364 au) compared to the CMC–AMPS hydrogel, attributed to the presence of N-fullerenes. The incorporation of N-fullerenes significantly enhanced the hydrogel’s antibacterial activity, increasing the inhibition zone against *E. coli* from 0 mm to 22 mm and against *S. aureus* from 15 mm to 24 mm. Molecular docking studies supported these findings, revealing shorter bond lengths between the hydrogel and bacterial proteins in the presence of N-fullerenes. Furthermore, the hydrogel displayed a unique “turn-on” fluorescence response upon interaction with bacteria, shifting from dark red to bright orange-red with *E. coli* and bright green with S. aureus, suggesting potential applications in bacterial detection and sensing. SEM analysis revealed a porous structure in both hydrogels, with the CMC–N-fullerene–AMPS hydrogel exhibiting a flowery morphology and pore sizes ranging from 377 to 931 µm due to the N-fullerenes, which is expected to contribute to enhanced interaction with the surrounding environment. These combined properties of enhanced antibacterial activity, fluorescence response, and porous structure suggest that the synthesized CMC–N-fullerene–AMPS hydrogel holds significant promise for applications in various fields, including biosensing in food packaging.

## 4. Materials and Methods

### 4.1. Materials

Sugarcane bagasse (SB) was obtained from the Quena Company for Paper Industry (Quena, Egypt). 2-Acrylamido-2-methyl-1-propanesulfonic acid (AMPS) was purchased from Alfa Aesar. N,N′-Methylenebisacrylamide (MBA) and potassium persulfate (KPS) were acquired from Sigma-Aldrich (Burlington, MA, USA). All chemicals and reagents were of analytical grade and used without further purification.

### 4.2. Preparation of Cellulose

Sugarcane bagasse (SB) was hydrolyzed by using 1.5% HCl at 120 °C for 2 h. The acid-treated SB was washed and then alkali-treated with 20 g NaOH in 300 mL water at 170 °C for 2 h to obtain pulp. Lignin was removed from 80 g of pulp by bleaching with 3% HClO_2_ and acetic acid at 80 °C for 2 h [65].

### 4.3. Microwave-Assisted Synthesis of Carboxymethyl Cellulose

An amount of 15 g of prepared cellulose was mixed with 30% NaOH solution and 18 g of monochloroacetic acid. This mixture was then subjected to microwave heating until complete dissolution. The synthesized CMC was then precipitated by adding 70% methanol, followed by filtration and oven drying [16,76].

### 4.4. Preparation of Nitrogen-Doped Fullerenes (N-fullerenes)

An amount of 1 g of CMC and 0.1 g of citric acid were dissolved in 20 mL of dimethylformamide. The mixture was microwave-irradiated at 100 °C and 750 W for 135 min using a Milestone Italy StartSynth microwave reactor (Pack2B Basic Single Vessel Kit, Milestone Inc., Bergamo, Italy) . The final solution was cooled to room temperature, centrifuged, filtered, and dried [38].

### 4.5. Preparation of Carboxymethyl cellulose–N-fullerene–g-poly(co-acrylamido-2-methyl-1-propane Sulfonic Acid)

An amount of 2 g of CMC was dissolved in 50 mL of distilled water at 60 °C with stirring. Then, 0.24 g of KPS diluted in 5 mL of distilled water was added. An aqueous suspension containing 2 g of fullerenes, 2 g of AMPS neutralized to pH 5.5 with NaOH, and 0.24 g of MBA was then added to the solution. The temperature was maintained at 65 °C for 10 min to initiate free radical polymerization on the CMC chains. Subsequently, the pH was adjusted to 5.5 and the temperature was set to 70 °C for 3 h. The resulting CMC–N-fullerene–AMPS hydrogel was washed with distilled water to remove any unreacted monomers, homopolymers, and crosslinkers. The hydrogel was then frozen at −80 °C for 3 h and freeze-dried. Another blank hydrogel was prepared without fullerenes and denoted as CMC–AMPS hydrogel.

### 4.6. Characterization

#### 4.6.1. Morphological Observations

Transmission electron microscopy (TEM) was performed using a JEOL JEM-2100 microscope (TEM, JEM-1230, Gunma, Japan) operating at 120 kV. Scanning electron microscopy (SEM) and energy-dispersive X-ray spectroscopy (EDX) were conducted with a Quanta/250-FEG scanning electron microscope (Thermo Fisher Scientific, Waltham, MA, USA) equipped with an EDX analyzer, using an acceleration voltage of 30 kV.

#### 4.6.2. Fluorescence Microscope

Fluorescence microscopy was carried out using a Jasco FP-6500 spectrofluorometer (Tokyo, Japan) equipped with a 150-watt xenon arc lamp.

#### 4.6.3. Fourier-Transform Infrared (FTIR) Spectra

Fourier-transform infrared (FTIR) spectra were acquired using a Mattson 5000 spectrometer (Unicam, UK) via the potassium bromide (KBr) disk method. The crystallinity index (LOI) and mean hydrogen bond strength (MHBS) were determined using Equations (1) and (2).

(1) $$\mathrm{LOI}=\frac{A_{1425}}{A_{900}}$$

(2) $$\mathrm{MHBS}=\frac{A_{OH}}{A_{CH}}$$

where A_1425_ and A_900_ refer to the FTIR absorbance at 1425 and 900 cm^−1^, respectively. In addition, A_OH_ and A_CH_ refer to the FTIR absorbance of the OH and CH peaks, respectively [66,94].

#### 4.6.4. DFT Calculations

Density Functional Theory (DFT) calculations were performed using Gaussian 09W at the B3LYP/6-31G(d) level of theory with Berny optimization. The investigated parameters included optimized geometries, ground state energies (E_T_), HOMO-LUMO energies (E_HOMO_, E_LUMO_, and E_g_), dipole moment (μ), absolute hardness (η), softness (σ, S), and additional electronic charge (ΔN_max_) [72,75].

(3) $$E_{gap}=(E_{LUMO}-E_{HOMO})$$

(4) $$\eta =\frac{\left(E_{LUMO}+E_{HOMO}\right)}{2}$$

(5) $$\sigma =\frac{1}{\eta }$$

(6) $$S=\frac{1}{2\eta }$$

(7) $$\Delta N_{max}=\frac{-Pi}{\eta }$$

#### 4.6.5. Swelling Behavior

The swelling behavior of the hydrogels was studied over time. A pre-weighed dry hydrogel sample was immersed in distilled water at room temperature. At designated intervals, the swollen hydrogel was removed and blotted dry with filter paper for 24 h to eliminate excess surface water. The swelling percentage was subsequently calculated using Equation (8).

(8) $$Swelling\left(\%\right)=\frac{W_{t}-W_{0}}{W_{0}}\times 100$$

where W_0_ and W_t_ stand for the hydrogel’s dry and swelling weights, respectively. For every hydrogel, the experiment was conducted three times [56].

## Figures and Tables

**Table 1:** The quantum chemical parameters of CMC, MBA, AMPS, CMC–AMPS hydrogel, N-fullerenes, and CMC–N-fullerene–AMPS hydrogel.

DFT B3LYP/6–31G (d)	CMC	MBA	AMPS	CMC–AMPS	N-Fullerenes	CMC–N-fullerene–AMPS
E_LUMO_ (eV)	−0.1232	−0.0196	0.0250	−0.0578	−0.1332	−0.1053
E_HOMO_ (eV)	−0.1650	−0.3027	−0.2713	−0.2033	−0.1755	−0.1924
E_g_ (eV)	0.0419	0.2831	0.2963	0.1454	0.0423	0.0871
E_T_ (au)	1434.557	−525.486	−939.989	−2741.402	−2290.040	−5158.364
μ (Debye)	11.141	3.781	9.219	24.309	1.334	41.321
ʷ (eV)	0.0209	0.1415	0.1481	0.0727	0.0211	0.0435
σ (eV)	47.732	7.0646	6.748	13.750	47.281	22.962
S (eV)	23.866	3.5323	3.374	6.875	23.640	11.481
ω (eV)	0.4955	0.0917	0.0511	0.1172	0.5632	0.2543

## Data Availability

The original contributions presented in the study are included in the article material; further inquiries can be directed to the author.
